# The past, present, and future of anti-idiotype antibodies

**DOI:** 10.3389/fimmu.2025.1686107

**Published:** 2025-11-17

**Authors:** William J. Murphy, Craig P. Collins, Heinz Kohler

**Affiliations:** 1 Departments of Dermatology and Internal Medicine, Hematology/Oncology, University of California (UC) Davis School of Medicine, Sacramento, CA, United States; 2 Department of Microbiology and Immunology, University of Kentucky, Lexington, KY, United States

**Keywords:** anti-idiotype antibodies, historical review and present situation, immunology, immunotherapy, immune regulation

## Abstract

Seminal discoveries led to the concept of the immune system as a complex network of antibodies and B-cells. In 1963, Kunkel et al. described individual antigenic specificities using antibodies directed against antibodies, hinting at the possibility of a network structure in the immune repertoire. In 1973, Jerne proposed the Network Theory that the immune system is a functional network of antibodies (idiotypes) and anti-idiotypic antibodies that are made in response due to the inherent immunogenicity of immunoglobulin variable chains. In 1974, anti-idiotypic responses were observed, providing proof of the Network Theory. In this review, the origin, as well as rise and fall, of idiotype research over the years is traced, citing examples of work that expanded the understanding of the network concept and its potential application. This includes broadly binding anti-idiotypic antibodies, anti-idiotype vaccines, anti-idiotypic antibodies as tools to trace monoclonal antibodies, and as immunotherapeutic biologicals. Future utility from using the Network Theory could involve cocktails of different monoclonal anti-idiotypic monoclonals. Studies can focus on how the Network Theory involves the generation of potential “antigen mirror” effects and how the network ultimately regulates both B and T cell responses over time. Despite the decline in popularity, aspects of the Network Theory are reemerging as evidence is generated on potential roles during host responses to pathogens or vaccines.

## The network hypothesis-early history

The immune Network Theory has been praised for its beauty and unique means in describing immune regulation, leading to the original popularity of the theory and the large amount of studies focusing on it. However, the subsequent clinical failure in using anti-idiotypic antibodies as therapeutics reduced its appeal over time and even led to skepticism regarding the legitimacy of the phenomenon, and the actual physiological relevance if indeed occurring. Initial attempts at clinical exploitation may have been hampered by perhaps an oversimplification of the Network Theory given the complex and diverse responses involved as well as the necessity of monoclonal antibodies to study it. As a result, the Idiotype Network theory is often not even mentioned in current immunology textbooks (1). Despite this skepticism, however, the theory has never been disproven, and a wide amount of research, both current and from the past, not only supports the Network Theory, but justifies its further investigation. This review will trace the beginning and development of the idiotype network concept, highlighting the vast amount of studies performed and the key findings that have relevance even today, as well as modern studies further evidencing the phenomenon and its biological importance.

In 1973, Niels Jerne proposed that the immune system is a network of antibodies and lymphocytes connected by protein complementarity regions (idiotype Greek ıδıoσ unique, eigen) on antibodies and antigen receptors that work in tandem to regulate the immune response (2). Jerne was intrigued by the huge number of different antibodies [10 (3)], each expressing specific idiotypic regions, and argued that antibodies can be immunogenic and potentially reciprocally recognize each other (2). The immune system is intricately regulated to achieve tolerance to “self.” For T cells, this occurs in the thymus during thymic education, being exposed to all the proteins encoded in the genome, with only those showing appropriate recognition passing the education process. However, variable-diversity-joining (VDJ) recombination, a unique process occurring in B and T lymphocytes, produces non-germline configuration B and T cell receptors (BCRs and TCRs), which generate completely novel proteins that have not seen by the immune system before. These therefore could and should be immunogenic, and capable of inducing immune responses towards them. The immune network proposed by Jerne prompted speculations of a functional regulatory idiotypic network of connected lymphocytes via antibodies.

Richter (4) proposed a network theory of the immune system, where the antigen-specific or idiotype response (Ab1) and the secondary anti-idiotype response (Ab2) are controlled by parameters of low and high zone tolerance. The quantitated tolerance differences would affect the network cascade involving each immunoglobulin, inducing further responses and counter-responses (Ab1-Ab2-Ab3 ….) Richter’s background as physicist enabled him to develop algorithms for a factional network. Similarly, another non-immunologist, Hoffman, a physicist, based his network theory on the role of T-cells in the immune network (5). T cells play a central role in antibody responses given their role in providing T cell “help” to the B cell and in isotype switching. TCRs also undergo gene rearrangement and conceivably can induce immunogenic responses in such a manner, albeit in the context of major histocompatibility complex (MHC).

By 1974, the rules and parameters of the immune system as a network were established, with Jerne’s landmark publication “Towards a Network Theory of the Immune System” generating interest, leading to his accreditation as the founder of the immune “Network Theory” (6). The Network Theory was based on several key discoveries, which, at first, dealt with polyclonal antibody responses against known antibodies.

Kunkel and colleagues (7) prepared antisera against isolated human antibodies and discovered individual antigenic specificities. However, it was the advent of monoclonal antibodies and use of inbred mouse models involving antibody responses to simple antigens such as haptens that really allowed determination and characterization of anti-idiotypic responses under more defined conditions. Using mouse models, Potter and Lieberman (8) identified individual antigenic determinants expressed by five of eight murine anti-phosphoryl-choline (PC) monoclonal antibodies derived from the responses, which were referred as T15. Mice were immunized to raised antibodies against T15 and antisera from these mice were then transferred to mice immunized with PC which then failed to respond to the PC antigen, indicating that anti-T15 antibodies were targeting PC antibodies (9). Similarly, *in vitro* B-cell cultures incubated with anti-T15 serum were specifically suppressed in PC responses (10). Follow-up studies (11) showed that neonatal mice injected with anti-T15 antibodies were clonally depleted (12) and unresponsive to PC for up to 9 months (13). The demonstration that antibodies against B cell expressed receptors caused their depletion led to a new therapeutic strategy to use anti-receptor antibodies to deplete B-cells (14, 15). Furthermore, these reports demonstrated that anti-idiotypic antibodies can influence the immune response, thereby suggesting the existence of a functional immune network. Related studies by Nisonoff and colleagues (16) reported that anti-idiotypic antibodies could induce suppression of the idiotypic specificities, indicating that B cell regulation across multiple antigen/antibody combinations existed.

Up to this point however, the immune Network Theory’s physiological relevance during an actual immune response to other more complex antigens was unclear. Anti-idiotypic antibodies can suppress a B cell response *in vitro* and can be generated *in vivo*, but whether an auto-anti-idiotypic response occurred during an actual immune response and had a similar effect, was not known. Kluskens and Kohler (17) found that mice immunized with the PC antigen developed phosphonyl idiotype positive anti-PC antibodies and antibodies that recognized the idiotype expressing anti-PC antibodies. Evidently, an Ab1-Ab2 symmetrical immune response occurred (18) that was part of a functional immune network, as predicted by Jerne. The Ab1-Ab2 pathway was observed in multiple other studies detecting auto-anti-idiotype antibodies together with antigen specific antibodies (19–23).

**Figure 1:**
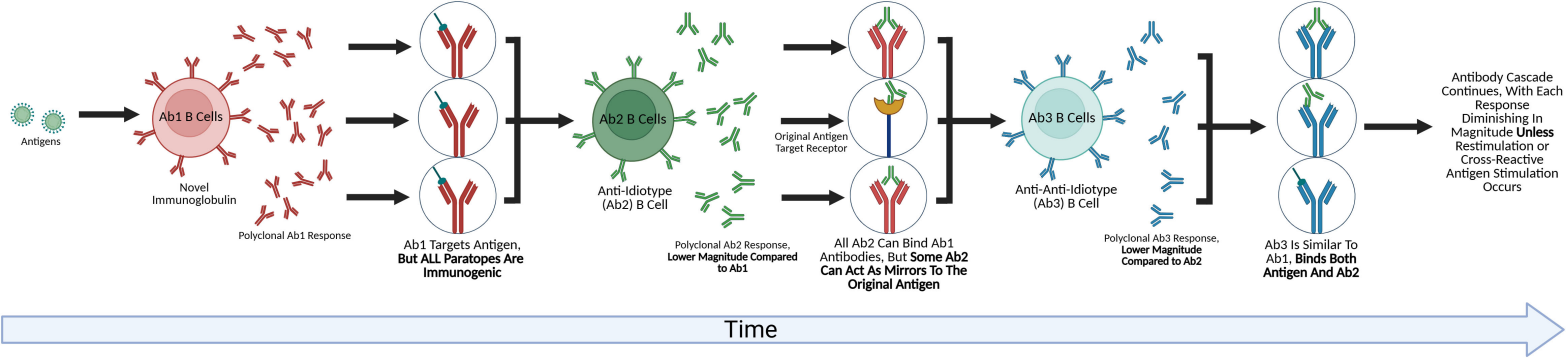
The future of idiotype network exploration. This figure depicts an anti-idiotype antibody cascade. B-cells responding to an antigen produce a novel antibody that has undergone VDJ rearrangement, or the Ab1 antibody. Because this antibody is novel to the host, a symmetrical Ab2 anti-idiotypic response is mounted against it. While all of the Ab2 antibodies are capable of binding the Ab1 and Ab1 producing B cells, some Ab2 paratopes have the potential to target the original targets of the antigen that triggered the Ab1 response, such as receptors. The Ab2 response also triggers an Ab3 response, in which all Ab3 can bind the Ab2, with some also having the potential of binding the original antigen that caused the cascade. Each wave of the cascade is of a lower magnitude than the previous, with Cazenave (24) demonstrating a 10-100x lower titer for the Ab2 onwards, eventually becoming undetectable, though chronic stimulation or repeated restimulation could amplify each wave of the cascade (25).

Jerne’s theory also proposed the existence of an “Internal Image’ of the antigen expressed by some of the anti-idiotype antibodies represent a “mirror” to the antigenic determinant being bound. Some of these “mirror” (Ab2) immunoglobulin mimic the original antigenic determinant (**Figure 1**). He coined these anti-idiotypes Ab2α and Ab2β. The binding of Ab2β to Ab1 is inhibited by the nominal antigen, but Ab2α is not. Thus, Ab2β can appear like the original antigen, and thus could be used as a surrogate vaccine. Several preclinical studies confirmed this vaccine concept using monoclonal Ab2 in inbred mice in infectious disease models. In addition, there are data indicating that some Ab2 induced by pathogens can by themselves induce pathogenic effects in recipients mirroring the original pathogen (25, 26). These studies provided proof that Ab2 can not only occur that mimic the original antigen but also can exert functions/effects that the original antigen has which has significant potential implications in infectious disease pathobiology, either from infection or vaccine responses depending on the antigen used.

However, there was debate regarding the occurrence and physiological relevance of such responses with the unique function of Ab2β as an internal antigen image being challenged by studies demonstrating that both Ab2α and Ab2β can induce antigen specific immune responses (27, 28). In addition, the concept of Ab2β as molecular mimic appeared incorrect base on structural analysis of a complex of Ab2β-with Ab1 (29). In another study comparing the complex of Ab1-antigen and Ab2 with antigen discovered that the chemistries were substantially different, although the surface area of both complexes were identical (30). Thus, the term “Network Antigen” was proposed (1, 3). To emphasize the role of the “network antigen” in the immune response, the term “regulatory idiotype” was introduced (31).

A role of T-cells in the immune network as suppressor T-cells induced by anti-idiotypic antibodies in the context of MHC, responsible for the maintenance of B-cell idiotype suppressed state using anti-idiotypic antibodies (32). The presence of idiotype suppressor T-cells was confirmed in a different antigen response (33). However, this was challenged (34) by showing that idiotype-negative T cell donors can support B-cells producing idiotype expressing antibodies suggesting that specific and non-antigen specific T cell responses may affect Ab2 responses.

In 1968, a report showed that anti-sera against IgM globulins that aggregate at a lower temperature, so-called cold agglutinins, reacted with other IgM preparations with the same activity, but not with IgM lacking this activity (35). Follow-up work confirmed the sharing of idiotypic markers with different monoclonal IgM antibodies (36). Using monoclonal anti-idiotypic antibodies against human anti-thyroglobulin (TG) detected the presence shared idiotypes in normal and disease-associated anti-TG autoantibodies (37). The so-called 16/6 idiotype is shared by anti-DNA autoantibodies in human and mice (38) is another example of idiotype sharing also demonstrating commonalities among species on this pathway.

## Application of the network theory in medicine

**Figure 2:**
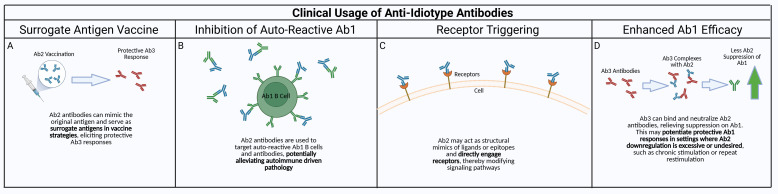
Potential clinical applications of anti-idiotype antibodies. Anti-idiotype antibodies have properties that could be exploited to produce viable clinical therapeutics. **(A)** Due to the anti-idiotype mirroring the Ab1 antibody, and thus the original target antigen of the antibody, a “surrogate” antibody vaccine utilizing Ab2 could be used to generate protective responses without exposing the patient to the actual antigen. **(B)** Autoreactive Ab1/Ab1-producing B cells could be targeted by using Ab2 against them, alleviating autoreactive driven immune pathology. This could also be taken a step further with Ab3 antibodies against autoreactive Ab2 antibodies, since Ab2 could have the ability to bind the host receptors that would be targeted by the original antigen. **(C)** Ab2 can also be used for receptor triggering when pathway activation is therapeutically beneficial. While many receptors are activated by ligand-induced dimerization, anti-idiotype antibodies can elicit or modulate receptor signaling through several alternative mechanisms, including higher-order receptor clustering, stabilization of pre-existing receptor dimers, FcγR-mediated crosslinking, or allosteric conformational changes that bias signaling without forming dimers (39). **(D)** Ab2 hinder Ab1 antibody responses – this can be detrimental if the Ab2 response is overamplified in conditions such as chronic stimulation/inflammation or repeated restimulation. This could be mediated by using Ab3 antibody against the Ab2, enhancing the Ab1 response in the absence of Ab2 suppression.

As mentioned previously, there have been multiple attempts to exploit anti-idiotype responses clinically but potentially more opportunities exist. This could occur via several pathways. The use of Ab2beta antibodies as surrogate antigens for vaccines, including potentially cancer vaccines, has been described (**Figure 2**), although the extreme specificity of each Ab2 antibody may necessitate a cocktail of mAbs instead of only one to elicit an efficacious robust and broad response. The demonstration that some Ab2 in some infectious disease models can trigger similar functional effects as the antigen indicates potential utility of antigen-mirror Ab2 beta properties where these antibodies can be used to bind and trigger cell surface receptors and mimic ligand effects similar to drugs.

Anti-idiotype responses have been exploited in cancer therapy as well. In 1982, monoclonal anti-idiotypic antibodies were used in passive immunotherapy of B-cell lymphoma (40). These non-Hodkin lymphomas expressed an idiotype, allowing Levy and colleagues to generate monoclonal anti-idiotypic antibodies targeting this idiotype. Subsequently, the patient was treated with the monoclonal directed against the lymphoma, with responses being observed (41). In follow-up clinical trials, the lymphoma idiotypes were used as a personalized vaccine with anti-tumor responses being reported (42, 43). These reports of using heterologous anti-idiotypic antibodies or vaccines generated from them indicated that potential clinical exploitation may be possible, although it would have to be tailored to the individual’s specific cancer and idiotype. One potential negative aspect of idiotype responses may affect current chimeric antigen receptor (CAR) therapies due to Ab2 responses to the mAb domains used in the CAR receptor although the extensive, and immunosuppressive, cytoreductive conditioning currently used may lessen such responses. Nonetheless, anti-idiotypic responses may lessen efficacy when mAb-based therapies are employed and manipulating or suppressing the Ab2 response may allow for increased or more prolong efficacy.

There is also reason to target autoimmune B cells via this approach, although as opposed to lymphomas which are clonal, polyclonal responses would need to be targeted using a cocktail of Ab2 antibodies (1). It is interesting to speculate that one of the mechanisms by which intravenous immunoglobulin (IVIG) works, often applied in autoimmune conditions with varying efficacy, is through the presence of pre-existing Ab2 antibodies in the serum. As the over-arching role of the Network Theory is immune regulation in dampening Ab1 responses over time, it is tempting to speculate that further development of Ab2 for autoimmune application is feasible, as not only would the autoreactive Ab1 be neutralized, but also the autoreactive B cells themselves would be suppressed. An important caveat to the use of Ab2 is also the likelihood that they in turn can elicit anti-idiotypic responses (Ab3) which could limit efficacy over time.

Because of the targeting of unique markers on antibodies, anti-idiotypic antibodies were used to trace antibodies in tissue as an immunodiagnostic. For example, Haase et al (44) used a broadly binding ant-idiotypic antibody to mark antibodies against HIV-1 in tissue sections. For example, Haase et al (44) used a broadly binding anti-idiotypic antibody to mark antibodies against HIV-1 in tissue sections. These anti-idiotypic reagents recognize shared idiotopes within the paratopes of antigen-specific antibodies, allowing indirect visualization of idiotype-bearing B cells or antibody deposits even in the absence of detectable antigen. Functionally, such anti-idiotypes bind the variable region of the Ab1 without mimicking the original HIV-1 antigen, thereby revealing idiotype expression rather than serving as receptor agonists or antagonists. This approach illustrates how anti-idiotypic antibodies can be exploited as analytical probes of immune network topology rather than as therapeutic agents.

Anti-idiotypic antibodies were explored as therapeutic vaccines (45, 46) or studied as regulators of the immune response (47). The scope of therapeutic targets using the anti-idiotype mimetic include viral diseases (SARS-CoV-2 (48), influenza (30), HIV (49), RSV (50)), toxins (51), and autoimmune diseases (52–54). It is interesting that the concept of the internal image anti-idiotype has been challenged by proposing to use polyclonal anti-idiotypic antibodies as the basis of a vaccine, prophylactic and therapeutic, instead of monoclonal to stimulate or suppress specific immune responses in the immune network (1, 55).

Although early idiotype-based lymphoma vaccines established proof-of-concept, the clinical potential of anti-idiotype strategies in oncology has been re-examined in recent years. Notably, the racotumomab (Vaxira^®^/1E10) anti-idiotype vaccine, which mimics the ganglioside NeuGcGM3, reached phase III clinical evaluation in non-small cell lung cancer (NSCLC) and melanoma, demonstrating survival benefits in subgroups and robust induction of anti-ganglioside humoral responses (56, 57). A newer formulation, racotumomab-alum, continues to be assessed in combination with checkpoint inhibitors to improve immunogenicity and antigen spreading. Parallel efforts include anti-idiotype antibodies mimicking disialoganglioside GD2 (TriGem/3F8-Id), evaluated in pediatric neuroblastoma and melanoma with evidence of long-lived anti-GD2 antibody and T-cell responses (58). Semisynthetic anti-idiotype peptibodies could also have potential, whereby short idiotype-binding peptides are fused to an Fc scaffold to engage both target BCRs and effector functions. Torchia et al. demonstrated that such peptibodies kill lymphoma cells in an idiotype-specific manner, induce macrophage phagocytosis, and eradicate human lymphoma in murine xenograft models (59). The modular peptide-Fc design enhances pharmacokinetics and effector cell recruitment while simplifying production relative to bespoke monoclonal antibodies (60). More broadly, the concept of linking tumor-targeting peptides to Fc domains to elicit ADCC has been explored in solid tumor models (61), providing proof of principle for the generalizability of peptide-Fc fusion strategies in cancer immunotherapy.

Recent studies have expanded the traditional concept of anti-idiotypic antibodies beyond passive regulation of B cell responses, demonstrating their potential to actively modulate receptor signaling. Kunze et al. engineered anti-idiotype nanobodies to tune synthetic cytokine receptor activation, showing that the interface between nanobody and receptor can be rationally designed to achieve graded receptor signaling, indicating that anti-idiotypic antibodies can serve as precise molecular tools to modulate receptor activity, not only as neutralizing or surrogate ligands but as tunable signaling modulators (62). Building on this concept, Wittich et al. developed a fully synthetic cytokine/receptor system using an engineered palivizumab IgG2 variant and anti-idiotype nanobodies targeting gp130 and Fas receptors. By combining antibody engineering with anti-idiotypic recognition, this approach allowed for controlled activation of receptor pathways relevant to immune regulation and cell fate decisions (63). Similarly, Ettich et al. demonstrated that palivizumab, an RSV-approved monoclonal antibody, could serve as a ligand in an anti-idiotype nanobody–based synthetic cytokine receptor system. This work illustrated the feasibility of converting existing therapeutic antibodies into tools for programmable receptor activation, thereby providing a translational bridge between classical anti-idiotypic theory and modern synthetic immunology applications (64). These studies underscore a potential role in which anti-idiotypic antibodies are not merely conceptual regulators within the idiotype network but can be exploited as engineered modulators of receptor-mediated signaling.

In the era of SARS-CoV-2, the concept of anti-idiotypic responses playing a role has resurfaced. While the use of monoclonal anti-idiotypic antibodies as surrogate vaccines has generally produced limited clinical efficacy in humans (1), a recent preclinical report (65) demonstrated that an anti-idiotypic monoclonal antibody derived from a neutralizing anti-spike antibody can act as an effective booster immunogen to enhance viral neutralization titers and T-cell responses. The study used K18-hACE2 to determine that administration of the anti-idiotype following primary mRNA vaccination increased neutralizing antibody titers approximately two- to three-fold relative to standard vaccine boost, with improved survival after live-virus challenge. It is important to note that responses in K18 mice may not be fully translational to human biology due to the overexpression/inappropriate tissue expression of hACE2 in K18 mice, so while these data could suggest that anti-idiotypic antibodies can amplify or shape existing immune memory, direct comparative clinical evidence versus licensed booster vaccines is limited. Interestingly, the anti-idiotypic monoclonal by itself does not induce an immune response, indicating that it is not an internal antigen or perhaps not sufficient or robust enough to induce a marked primary response. More work is needed on the extent where an Ab2 can induce protective Ab3 or boost an existing Ab1 responses. Future clinical applications of anti-idiotype and anti-anti-idiotype antibodies are described in **Figure 2**.

As discussed earlier, anti-idiotypic responses have the capability of inhibiting protective idiotype Ab1 responses. In the case of CoV2 vaccine responses, it is interesting to speculate that the relatively short half-life of protective anti-Spike response may be due to later suppression by Ab2 responses (66–68). Given the strong homology between CoV2 and other endemic coronaviruses causing common upper respiratory tract infections suggests that cross-reactive secondary responses to these endemic coronaviruses interfere and suppress CoV2 responses (69–72). An area of research that has not been pursued is possibility that cross-reactive anti-idiotype responses affect the maintenance of the protective response. It will be particularly important to study the need for repeated vaccine boosting to determine the role of Ab2 response in achieve protection.

Another interesting effect of the idiotypic network in the response to CoV2 relates to biological mimicry of auto-anti-idiotypic antibodies (Ab2) (73). The target antigen for CoV2 vaccines is the Spike protein, which, in turn, binds receptors such as ACE2 on cells for entry. Therefore, due to Ab2 having a potential antigen mirror, some Ab2 could also be able to bind ACE2 (25, 73, 74). It has been reported in patients with CoV2 infection that anti-ACE2 antibodies could be detected (74, 75). Studies in mice have also recently demonstrated that purified Spike protein (76) or CoV2 vaccine application results in anti-ACE2 antibody production (25). The potential effects of such Ab2 antibodies could both agonistic by triggering ACE2 or antagonistic by blocking or depleting ACE2^+^ cells, thereby augmenting the angiotensin response (77). It has been postulated (54) that anti-idiotypic antibodies are involved in mechanisms in persistent or chronic pathologies following CoV2 infection resolution. Animal models of immune dysfunction are needed to dissect the role of Ab2 responses in autoimmune pathologies.

## Conclusion

Idiotype research with over 5000 published data spanning a time of more than 60 years were scanned to trace the development of the Idiotype Network theory, to highlight important discoveries on the function of the immune system and to evaluate its impact on immunology in general. In 1963 antibodies against antibodies were determined to exist. These anti-antibodies detected antigenic targets that were specific for each antibody representing individual determinants. A few years later these anti-antibodies were idiotypic and used to probe murine myeloma proteins. Three out of five myelomas expressed the same idiotype, indicating sharing of idiotypic markers suggesting that anti-idiotypic antibodies are potential tools in immunodiagnostics. Furthermore, idiotypic antibodies can suppress an immune response and induce clonotypic B-cell depletion potentially regulating autoreactive B cell clones (**Figure 2**).

In 1974 the Immune Network theory was announced, supported by analytical network models. Proof that idiotype-anti-idiotype responses occur during an immune response showed that the immune system is a network (7). Numerous studies using inbred animals exemplified the role of the idiotypic network. While clinical trials with monoclonal anti-idiotypic antibodies failed (1, 78), the need was expressed that polyclonal or oligoclonal therapeutic anti-idiotype antibodies, mimicking the polyclonal nature of the network, could be effective in therapy and as vaccines. A challenge for idiotype research is the mechanism of autoimmunity induced by off-target immune response such as Guillain-Barre Syndrome (79, 80). A recent report (81) on the Idiotype Network in anti-SARS-CoV-2 immune response highlights the need to further focus on the immune network in the absence of evidence for molecular mimicry. Experimentally induced off-target immune reactions are first steps to dissect immune network dysfunction (82). Clearly, more mechanistic preclinical and clinical work assessing the role and effects of anti-idiotype responses are needed to better understand it and perhaps clinically exploit it.
